# Are pregnant women prioritized for bed nets? An assessment using survey data from 10 African countries

**DOI:** 10.9745/GHSP-D-14-00021

**Published:** 2014-05-13

**Authors:** Emily Ricotta, Hannah Koenker, Albert Kilian, Matthew Lynch

**Affiliations:** a Johns Hopkins Bloomberg School of Public Health, Center for Communication Programs, Baltimore, MD, USA.; b Tropical Health LLP, Montagut, Girona, Spain.

## Abstract

**Background:**

Malaria in pregnancy is a major public health concern, contributing to roughly 11% of neonatal deaths and to 25% of all maternal deaths in some parts of the world. The World Health Organization has recommended priority interventions for malaria during pregnancy, including use of insecticide-treated nets (ITNs), but net distribution has shifted recently to a universal coverage paradigm rather than one targeting vulnerable populations.

**Methods:**

To determine whether and to what extent pregnant women are prioritized within the household for ITN use, we assessed national survey data from 2009–2013 in 10 African countries. Proportion of pregnant women who slept under an ITN the previous night and 95% confidence intervals were calculated and compared between countries. Within-country logistic regression examined whether pregnancy was significantly associated with ITN use the previous night compared with other risk groups, and the predicted probability of net use for each risk group was calculated holding other covariates constant.

**Results:**

A median 58% of households reported owning at least 1 ITN. On average, across all 10 countries, 35% of pregnant women in households with at least 1 ITN used a net. Households with universal coverage (at least 1 ITN per 2 people) had higher levels of net use among all family members; for example, 79% of pregnant women, on average, used a net in such households. In all countries, the predicted probability of ITN use by pregnant women was significantly higher than the probability of net use by most other household members except non-pregnant women of reproductive age.

**Conclusion:**

These findings suggest that both pregnant women and non-pregnant women of reproductive age are being prioritized within the household for net use. However, behavior change communication strategies are needed to achieve ITN use goals for pregnant women.

## Abstract (precis)

Women of reproductive age are generally more likely to sleep under an insecticide-treated net (ITN) than other household members. Universal coverage increases ITN use by all family members, including pregnant women. However, BCC efforts are needed to achieve desired levels of bed net use, which is especially important for pregnant women.

## INTRODUCTION

Malaria in pregnancy is a major public health concern, contributing to high rates of maternal and newborn morbidity and mortality. More than 30 million pregnant women reside in malaria-endemic areas of Africa, where *Plasmodium falciparum* infection in pregnancy is associated with low neonatal birth weight and where the infection contributes to roughly 11% of neonatal deaths.^1^ One of the most serious and potentially deadly consequences of malaria during pregnancy is maternal anemia, which presents even in women with asymptomatic malaria infection and in those with undetectable infection by blood smear due to mass sequestration in the placenta.^2^ In some parts of the world, malaria infection during pregnancy contributes directly to 25% of all maternal deaths.^3^

Malaria infection during pregnancy contributes to 25% of all maternal deaths in some parts of the world.

Because of the significant impact of this disease on pregnant women and newborns, in 2000, multiple African countries south of the Sahara pledged to fight malaria in pregnancy with insecticide-treated nets (ITNs), intermittent preventive treatment of malaria in pregnancy (IPTp), and improved case management.^4^ Three years later, the World Health Organization released “A Strategic Framework for Malaria Prevention and Control During Pregnancy in the African Region” to develop a standardized method for preventing and controlling malaria during pregnancy.^5^ By 2007, 39 African countries had incorporated part, or all, of this framework into their national malaria control plans.^6^ However, the extent to which these new strategies have been deployed or have reduced maternal malaria is uncertain.

According to an analysis by Stenberg and colleagues (2013), scaling up ITN coverage and IPTp and ensuring availability of appropriate malaria treatment could prevent 153 million episodes of malaria by 2035, saving US$5 billion in drug costs alone and US$280 million in outpatient visits.^7^ Additionally, studies have shown that targeting pregnant women and infants during ITN distribution campaigns is an effective method of reducing all-cause post-neonatal mortality.^8^^–^^11^

Timing of ITN use during pregnancy is important. While the effects of malaria infection in the first trimester are largely unknown, risk of miscarriage seems to increase compared with women who become infected later in pregnancy, especially among women who develop clinical malaria.^12^ The highest rates of infection, severe disease, and fetal impairment occur in the second and third trimesters, but IPTp cannot be given to pregnant women in the first trimester due to potential teratogenicity and possible fetal death.^12^^,^^13^ These factors make it important for women to attend antenatal care (ANC) to obtain an ITN early during pregnancy.^3^^,^^12^^,^^14^

Since 2009, there has been a move away from prioritizing vulnerable populations (such as children under 5 and pregnant women) during net distribution to a universal coverage paradigm.^15^ These campaigns strive to provide every household with 1 net per 2 people, or 1 net per sleeping space, regardless of household composition (that is, number of pregnant women and children). This strategy allows countries to rapidly attain high levels of net coverage throughout a region and includes households that do not have a pregnant woman or young children; however, in order to maintain protection across the population, there needs to be additional, focused distribution targeting pregnant women and children to ensure ITN coverage at critical fetal and infant growth stages.^16^ Within the context of universal coverage campaigns, it is unclear whether pregnant women continue to be prioritized for ITN use, in terms of nets allocated at the national level that are specifically targeted for ANC distribution as well as net allocation within the household.

In this article, we focus on assessing available survey data from 10 countries in Africa to determine whether and to what extent pregnant women are prioritized within the household for ITN use. By applying the newly recommended indicators of access to an ITN within the household to this group, it is possible to see more precisely where pregnant women are using nets.^17^ These data provide a better understanding of net use behavior, differentiating between those who do not use a net because they do not have access to one versus those who have access to a net and do not use one for behavioral reasons.

## METHODS

We analyzed recent Demographic and Health Surveys (DHS) and Malaria Indicator Surveys (MIS) collected in 2009 or later from 10 African countries (Table 1). Criteria for selection of the datasets included availability of data on individual ITN use and whether there were any pregnant women on the day of the survey within the household. The most recent publicly available dataset from each country was chosen; 9 of 10 datasets were from 2010–2013, to reflect recent scale up of coverage of long-lasting insecticidal nets (LLINs). All datasets were obtained with permission from MEASURE DHS.

**TABLE 1.** Characteristics of Surveys Included in the Analysis and Key Indicators Related to Malaria in Pregnancy

					**Household ITN Ownership % (95% CI)**	
**Country**	**Survey Type**	**Year of Survey**	**No. of Households Sampled**	**Pregnant Women % (95% CI)**	**Partial Coverage^a^**	**Universal Coverage^b^**
Liberia	MIS	2011	4,162	9.0 (8.1–10.0)	55.1 (54.3–55.8)	9.4 (8.9–9.8)
Madagascar	MIS	2013	8,574	7.4 (6.8–8.0)	62.4 (61.9–62.9)	19.5 (19.1–19.9)
Malawi	DHS	2010	24,825	9.3 (9.0–9.7)	60.4 (60.2–60.7)	14.7 (14.5–14.9)
Mozambique	DHS	2011	13,919	10.2 (9.7–10.7)	53.6 (53.2–54.0)	16.6 (16.3–16.9)
Nigeria	MIS	2010	5,895	10.8 (10.1–11.6)	48.8 (48.2–49.3)	9.8 (9.5–10.2)
Rwanda	DHS	2010	12,540	6.8 (6.4–7.3)	85.8 (85.5–86.1)	32.8 (32.4–33.2)
Senegal	DHS	2010	7,902	8.4 (8.0–8.9)	75.5 (75.2–75.8)	14.3 (14.1–14.6)
Tanzania	THMIS	2011	10,040	9.3 (8.8–9.8)	92.0 (91.8–92.2)	41.2 (40.8–41.6)
Uganda	MIS	2009	9,033	9.8 (9.0–10.8)	51.2 (50.5–51.9)	12.0 (11.6–12.4)
Zimbabwe	DHS	2010	9,756	7.8 (7.3–8.4)	30.4 (29.9–30.8)	9.2 (9.0–9.5)

Abbreviations: DHS, Demographic and Health Survey; ITN, insecticide-treated net; MIS, Malaria Indicator Survey; THMIS, Tanzania HIV/AIDS and Malaria Indicator Survey.

a At least 1 ITN per household.

b At least 1 ITN per 2 people.

Analyses were completed using R: A Language and Environment for Statistical Computing, version 3.0.2 (R Foundation for Statistical Computing, Vienna, Austria), and Stata Statistical Software: Release 13 (StataCorp LP, College Station, TX). Proportion of pregnant women who slept under an ITN the previous night and 95% confidence intervals were calculated and compared between countries. We used within-country logistic regression to examine whether pregnancy was significantly associated with ITN use the previous night when compared with other risk groups, including children under 5, children 5–14 years old, non-pregnant women of reproductive age (WRA), men ages 15–49 years, and adults over 49 years old. This analysis was restricted to those households that reported owning at least 1 ITN (referred to here as partial household coverage).

Covariates were selected for each country using *a priori* research and included the categorical risk group variable, residence (urban/rural), administrative divisions, month the survey was conducted, and wealth quintiles. Wealth was based on household assets and obtained by principle component analysis.^18^

Additional indicators used for analysis were developed by the Roll Back Malaria (RBM) Monitoring and Evaluation Reference Group,^17^ which included the “proportion of households with at least 1 ITN for every 2 people” to act as a measure for sufficient nets within the household so that every member could have used a net (referred to as universal household coverage).

The outcome variable was individual ITN use the prior night (yes/no), and *P* values < .05 were considered significant. Following regression, the predicted probability of net use for each risk group was calculated, holding other covariates constant at their observed value.

## RESULTS

The number of households surveyed ranged from 5,895 (Nigeria) to 24,825 (Malawi). The median proportion of those surveyed who were pregnant was 9%, ranging from 7% (Madagascar and Rwanda) to 11% (Nigeria) (Table 1).

### Household ITN Ownership

The median proportion of households reporting ownership of at least 1 ITN was 58%. This proportion varied widely by country, from 30% in Zimbabwe to 92% in Tanzania (Table 1). Universal net coverage (at least 1 ITN per 2 people) was lower, ranging from 9% in Liberia and Zimbabwe to 33% in Rwanda.

### ITN Use by Pregnant Women

Proportion of net use by pregnant women varied widely by country. Among households with partial net coverage, the proportion ranged from 5.5% in Zimbabwe to 60% in Rwanda. Households with universal net coverage had higher net use; the proportion of net use by pregnant women in these households ranged from 53% in Zimbabwe to 91% in Madagascar and Uganda (Table 2). Mean proportion of net use by pregnant women across all countries in households with at least 1 ITN was 35%, compared with 79% among households with universal net coverage.

**TABLE 2.** ITN Use Among Pregnant Women, Non-Pregnant Women of Reproductive Age, and Other^a^ Household Members, by ITN Household Coverage

**Country and Risk Group**	**Partial Coverage Households^b^ % (95% CI)**		**Universal Coverage Households^c^ % (95% CI)**	
Liberia				
Pregnant	38.9 (32.7, 45.5)		86.2 (67.4, 95.5)	
WRA	36.2 (34.3, 38.1)		79.5 (74.3, 83.8)	
Other	25.5 (24.8, 26.2)		74.9 (72.5, 77.2)	
Madagascar				
Pregnant	40.6 (36.1, 45.3)		91.1 (85.3, 94.9)	
WRA	41.0 (39.8, 42.3)		87.6 (85.9, 89.0)	
Other	36.9 (36.3, 37.5)		85.8 (84.8, 86.7)	
Malawi				
Pregnant	28.2 (26.2, 30.4)		72.7 (68.2, 76.7)	
WRA	28.5 (27.8, 29.2)		71.1 (69.6, 72.5)	
Other	20.5 (20.2, 20.8)		65.8 (64.9, 66.6)	
Mozambique				
Pregnant	22.6 (20.2, 25.3)		68.9 (63.6, 73.8)	
WRA	22.8 (22.0, 23.6)		68.8 (66.8, 70.7)	
Other	18.4 (18.1, 18.8)		64.2 (63.1, 65.2)	
Nigeria				
Pregnant	26.6 (23.1, 30.4)		81.2 (71.7, 88.2)	
WRA	23.0 (21.9, 24.2)		69.8 (66.0, 73.2)	
Other	17.8 (17.3, 18.3)		69.4 (67.4, 71.3)	
Rwanda				
Pregnant	60.0 (55.3, 64.5)		86.8 (83.1, 89.9)	
WRA	58.2 (56.9, 59.4)		83.3 (82.0, 84.5)	
Other	43.3 (42.7, 43.8)		76.1 (75.4, 76.8)	
Senegal				
Pregnant	37.2 (34.4, 40.1)		73.7 (67.4, 79.2)	
WRA	33.8 (33.0, 34.6)		72.6 (70.7, 74.6)	
Other	28.5 (28.1, 28.9)		65.1 (64.1, 66.1)	
Tanzania				
Pregnant	56.5 (52.4, 60.5)		84.9 (81.2, 88.0)	
WRA	55.6 (54.3, 56.8)		83.9 (82.7, 85.0)	
Other	50.8 (50.2, 51.4)		77.5 (76.9, 78.2)	
Uganda				
Pregnant	32.8 (27.4, 38.8)		90.7 (77.0, 97.0)	
WRA	27.2 (25.4, 29.1)		82.4 (78.2, 85.9)	
Other	17.4 (16.8, 18.1)		74.0 (72.0, 76.0)	
Zimbabwe				
Pregnant	5.5 (3.9, 7.6)		52.6 (41.0, 63.9)	
WRA	6.5 (6.0, 7.1)		46.7 (43.3, 50.1)	
Other	4.9 (4.6, 5.1)		39.9 (38.2, 41.8)	

Abbreviations: ITN, insecticide-treated net; WRA, (non-pregnant) women of reproductive age.

a “Other” category comprises children through age 14, men ages 15–49, and adults over age 49.

b At least 1 ITN per household.

c At least 1 ITN per 2 people.

Households with universal coverage had higher average bed net use among pregnant women than households with partial coverage: 79% vs. 35%, respectively.

### ITN Use by Pregnant Women Versus Other Household Members

Net use was generally higher among pregnant women and non-pregnant WRA than among other household members. There was no significant difference in use between pregnant women and WRA in any country, regardless of whether the household had sufficient nets for the entire family. In households with at least 1 ITN, a significantly higher proportion of pregnant women than “other” household members (comprising children through 14 years of age, men ages 15–49 years, and adults over 49 years) used nets in 8 of the 10 countries included in the analysis (Liberia, Malawi, Mozambique, Nigeria, Rwanda, Senegal, Tanzania, and Uganda). In households with universal coverage, a significantly higher proportion of pregnant women than other household members used nets in Malawi, Rwanda, Senegal, Tanzania, and Uganda (Table 2).

**FIGURE 1.**
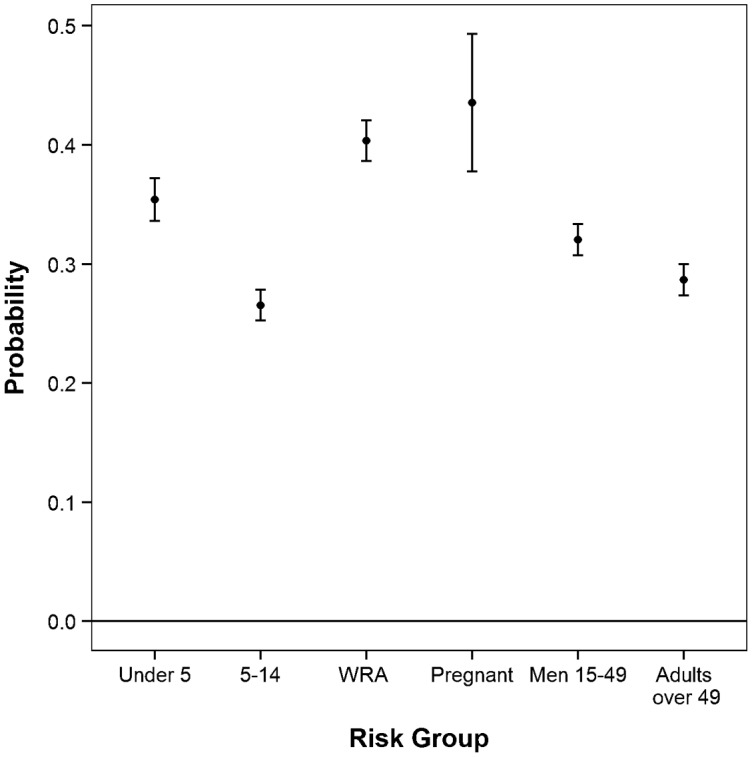
Adjusted Probability of ITN Use by Risk Group, Liberia, 2011 Abbreviations: ITN, insecticide-treated net; WRA, (non-pregnant) women of reproductive age. Pregnant women were significantly more likely to use an ITN than children under 5, children ages 5–14, men ages 15–49, and adults over 49 years. ITN use by pregnant women was *not* significantly different than use by non-pregnant WRA. Error bars indicate 95% confidence intervals.

**FIGURE 2.**
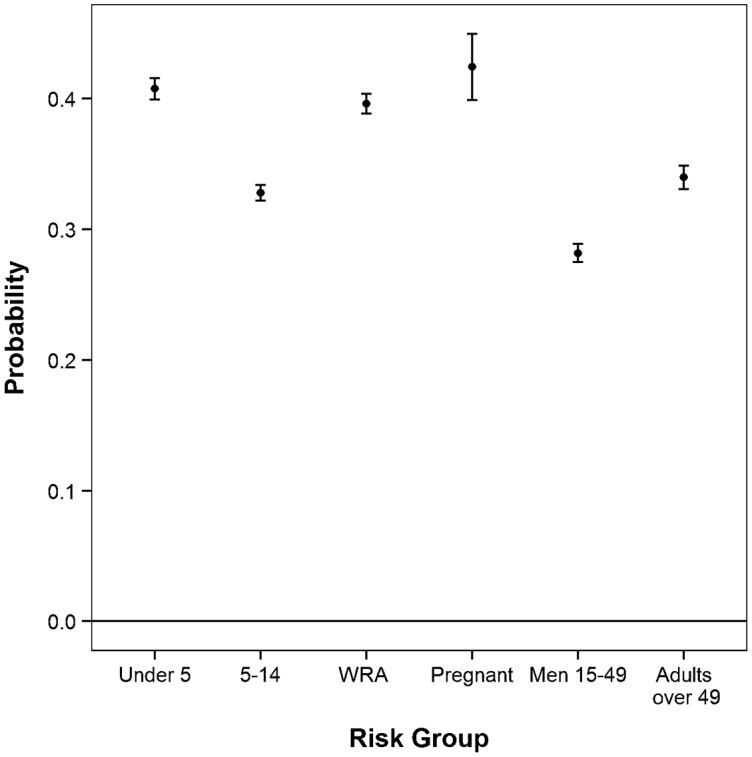
Adjusted Probability of ITN Use by Risk Group, Senegal, 2010 Abbreviations: ITN, insecticide-treated net; WRA, (non-pregnant) women of reproductive age. Pregnant women were significantly more likely to use an ITN than children ages 5–14, men ages 15–49, and adults over 49 years. ITN use by pregnant women was *not* significantly different than use by non-pregnant WRA or children under 5. Error bars indicate 95% confidence intervals.

In all countries, the predicted probability of ITN use by pregnant women was significantly higher than the probability of ITN use by children ages 5–14, men ages 15–49, and individuals over 49 after controlling for other household variables. See, for example, Figure 1, showing data from Liberia. (For data from other countries, see Supplemental Figures 1–5). Pregnant women were also more likely to use an ITN than children under 5 in all but 4 countries (Nigeria, Senegal, Tanzania, and Zimbabwe). See Figure 2 for sample data from Senegal and Supplemental Figures 6–8 for data from Nigeria, Tanzania, and Zimbabwe. In all countries, ITN use by pregnant women was *not* significantly different than for non-pregnant WRA when controlling for other household variables.

Pregnant women and non-pregnant women of reproductive age were significantly more likely to use a bed net than other household members.

## DISCUSSION

In this study, we compared ITN use by pregnant women in households with and without universal net coverage (that is, 1 net per 2 people) to determine whether lack of household access to ITNs was the main reason for non-use of nets by pregnant women. The results of this study demonstrate that across the 10 study countries, pregnant women as well as non-pregnant women of reproductive age were more likely to have used an ITN the night before the survey than other household members. In addition, an even higher proportion of pregnant women who lived in a household with universal coverage than pregnant women in households without universal coverage used nets. This suggests that both non-pregnant women of reproductive age and pregnant women are being prioritized within the household and that non-use is indeed more related to lack of access to an ITN rather than due to a behavioral issue.

Lack of access to bed nets is an important barrier to use of nets by pregnant women.

Greater access to nets, however, does not always translate into greater use by pregnant women; behavior change communication (BCC) is still needed to ensure prioritization of and use by pregnant women. In some countries that recently had a net distribution campaign (such as in Madagascar and Tanzania, which had major distributions between 2009–2010 and 2010–2011, respectively), households were more likely to report higher net ownership, thus reducing the use:access gap.^19^ But these countries were not always the most likely to prioritize pregnant women, and some countries with low universal net coverage rates are more likely to prioritize pregnant women for net use. For example, in Nigeria only 9.8% of households reported having universal coverage, yet pregnancy was a significant predictor of net use when compared with children ages 5–14, men ages 15–49, and individuals over 49. This demonstrates that pregnant women were prioritized at the net distribution level, the household-allocation level (which also includes prioritization of non-pregnant women of reproductive age), or both. In early 2009, Nigeria embarked on a universal coverage campaign to distribute LLINs and spread messaging about malaria prevention, including in ANC clinics.^20^ Such BCC messages promoting ITN use for pregnant women have been part of national communication strategies since 2003,^5^ and it appears that households and communities have internalized them.^21^

This study is not the first to note prioritization of ITN use by pregnant women in recent years. Data from as early as 2004 demonstrate that children and women of reproductive age were more likely to use ITNs, especially in households with more nets per people, in African countries including Tanzania and Zambia.^21^^,^^22^ Additionally, these nets were in better condition than those used by older children and adults.^21^ In contrast, data from 15 national surveys between 2003–2006 showed that pregnant women had less access to ITNs than other household members and were not prioritized within the household.^23^

The upward trend has led to an overall increase in net use by pregnant women, which can be attributed to increased intra-household net access as well as prioritization of all women of reproductive age by those household members responsible for net use and allocation decisions. In some countries evaluated in this study, net use by pregnant women has exceeded RBM's 2010 goal of 80% use in households that report universal coverage.^11^^,^^24^ However, in households owning less than 1 net per 2 people, no countries met this goal.

Insufficient access to nets for pregnant women can be due to many factors, including local- and national-level stockouts of ITNs at ANC clinics, delays in ANC attendance, and insufficient provider training.^11^^,^^25^ These problems call for additional monitoring and evaluation of the supply chain and accountability both nationally and locally as well as improving ANC distribution systems to ensure consistent access to nets by pregnant women.

Supply chains for bed nets should be monitored to ensure consistent access to nets by pregnant women.

### Limitations

One limitation of this study is that in some countries net ownership and use was so high that the difference in net use between pregnant and non-pregnant household members was too small to detect. In Tanzania, over 90% of households reported having at least 1 bed net, and 41% of households had at least 1 net for 2 people (Table 1). However, the difference in net use between pregnant and non-pregnant individuals was only 1%–2%. Although this could reflect lack of prioritization, it is more likely due to a long-standing targeted distribution program through ANC services, a recent universal coverage campaign, high net use, and a small sample size of pregnant women. Another limitation of this study is that net use was not stratified geographically within each country. It is known that net use varies regionally,^26^ but there were not enough data in this study to evaluate these differences.

## CONCLUSION

Lack of access to bed nets appears to be the more important factor for non-use of ITNs by pregnant women in the 10 African countries analyzed in this study, rather than behavioral issues. However, it would be premature to assume that increasing net access alone would solve the problem completely, particularly since shortages and gaps in coverage are inevitable at national and local levels. Strategies such as behavior change communication to promote prioritization of pregnant women at both national program and household levels are necessary to achieve the coverage goals set by RBM for pregnant women. Such strategies can increase pregnant women's access to ITNs, further strengthen the culture of net use in countries in general, and continue to encourage net use specifically by pregnant women at all stages of pregnancy.

## References

[1] Guyatt HL Snow RW Impact of malaria during pregnancy on low birth weight in sub-Saharan Africa. Clin Microbiol Rev. 2004;17(4): 760–769 10.1128/CMR.17.4.760-769.2004 15489346 PMC523568

[2] Uneke CJ Impact of placental Plasmodium falciparum malaria on pregnancy and perinatal outcome in sub-Saharan Africa: I: introduction to placental malaria. Yale J Biol Med. 2007;80(2): 39–50 18160989 PMC2140183

[3] Schantz-Dunn J Nour NM Malaria and pregnancy: a global health perspective. Rev Obstet Gynecol. 2009;2(3): 186–192 19826576 PMC2760896

[4] World Health Organization (WHO), Roll Back Malaria Partnership Secretariat. The Abuja Declaration and the plan of action. Geneva: WHO/Roll Back Malaria; 2000 Available from: http://whqlibdoc.who.int/hq/2003/WHO_CDS_RBM_2003.46.pdf?ua=1

[5] World Health Organization (WHO). A strategic framework for malaria prevention and control during pregnancy in the African region. Brazzaville: WHO Regional Office for Africa; 2004 Available from: http://whqlibdoc.who.int/afro/2004/AFR_MAL_04.01.pdf?ua=1

[6] President's Malaria Initiative; Roll Back Malaria Partnership; Maternal and Child Health Integrated Program (MCHIP). Malaria protection in pregnancy: a lifesaving intervention for preventing neonatal mortality and low birth weight. Washington (DC): MCHIP; 2012 Available from: http://www.mchip.net/node/1501

[7] Stenberg K Axelson H Sheehan P Anderson I Gulmezoglu AM Temmerman M Advancing social and economic development by investing in women's and children's health: a new Global Investment Framework. Lancet. 2014;383(9925): 1333–1354 10.1016/S0140-6736(13)62231-X 24263249

[8] Hawley WA ter Kuile FO Steketee RS Nahleen BL Terlouw DJ Gimnig JE Implications of the western Kenya permethrin-treated bed net study for policy, program implementation, and future research. Am J Trop Med Hyg. 2003;68(4 Suppl): 168–173 12749501

[9] ter Kuile FO Terlouw DJ Phillips-Howard PA Hawley WA Friedman JF Kariuki SK Reduction of malaria during pregnancy by permethrin-treated bed nets in an area of intense perennial malaria transmission in western Kenya. Am J Trop Med Hyg. 2003;68(4 Suppl): 50–60 12749486

[10] Feng G Simpson JA Chaluluka E Molyneux ME Rogerson SJ Decreasing burden of malaria in pregnancy in Malawian women and its relationship to use of intermittent preventive therapy or bed nets. PLoS One. 2010;5(8): e12012 10.1371/journal.pone.0012012 20700457 PMC2917365

[11] van Eijk AM Hill J Larsen DA Webster J Steketee RW Eisele TP Coverage of intermittent preventive treatment and insecticide-treated nets for the control of malaria during pregnancy in sub-Saharan Africa: a synthesis and meta-analysis of national survey data, 2009-11. Lancet Infect. Dis. 2013;13(12): 1029–42 10.1016/S1473-3099(13)70199-3 24054085

[12] Desai M ter Kuile FO Nosten F McGready R Asamoa K Brabin B Epidemiology and burden of malaria in pregnancy. Lancet Infect Dis. 2007;7(2): 93–104 10.1016/S1473-3099(07)70021-X 17251080

[13] World Health Organization (WHO). WHO policy brief for the implementation of intermittent preventive treatment of malaria in pregnancy using sulfadoxine-pyrimethamine (IPTp-SP). Geneva: WHO; 2013 (rev 2014 Jan) Available from: http://www.who.int/malaria/publications/atoz/policy_brief_iptp_sp_policy_recommendation/en/

[14] Desai M Dellicour S Effects of malaria and its treatment in early pregnancy. Lancet Infect Dis. 2012;12(5): 359–360 10.1016/S1473-3099(11)70345-0 22169410

[15] Roll Back Malaria, Malaria in Pregnancy Working Group. Consensus statement: optimizing the delivery of malaria-in-pregnancy interventions. Geneva: WHO/Roll Back Malaria; 2013 Available from: http://www.mhtf.org/wp-content/uploads/sites/17/2012/05/MiP_1_V8_Final.pdf

[16] WHO Malaria Policy Advisory Committee and Secretariat. Malaria Policy Advisory Committee to the WHO: conclusions and recommendations of September 2013 meeting. Malar J. 2013;12:456 10.1186/1475-2875-12-456 24359206 PMC3896675

[17] MEASURE Evaluation; MEASURE DHS; President's Malaria Initiative; Roll Back Malaria Partnership; UNICEF; World Health Organization. Household survey indicators for malaria control. Chapel Hill, NC: MEASURE Evaluation; 2013 Available from: http://www.rollbackmalaria.org/toolbox/docs/rbmtoolbox/tool_HouseholdSurveyIndicatorsForMalariaControl.pdf

[18] Vyas S Kumaranayake L Constructing socio-economic status indices: how to use principal components analysis. Health Policy Plan. 2006;21(6): 459–468 10.1093/heapol/czl029 17030551

[19] Koenker H Kilian A Recalculating the net use gap: a multi-country comparison of ITN use versus ITN access. PLoS One. Forthcoming 2014 10.1371/journal.pone.0097496 PMC4030003 24848768

[20] Malaria Consortium [Internet]. London: Malaria Consortium; c2014 Managing the risk of malaria in pregnancy in Nigeria; 2012 Apr 18 [cited 2014 Jan 6]; [about 2 screens]. Available from: http://www.malariaconsortium.org/news-centre/managing-the-risk-of-malaria-in-pregnancy-in-nigeria.htm

[21] Tsuang A Lines J Hanson K Which family members use the best nets? An analysis of the condition of mosquito nets and their distribution within households in Tanzania. Malar J. 2010;9:211 10.1186/1475-2875-9-211 20663143 PMC2918626

[22] Baume C Marin C Payes R Intra-household use of mosquito nets: who sleeps under the net? Washington (DC): Academy for Educational Development, NetMark; 2004 Available from: http://pshi.fhi360.org/pdfs/Intra-Household_Use_of_Mosquito_Nets.pdf

[23] Eisele TP Keating J Littrell M Larsen D Macintyre K Assessment of insecticide-treated bednet use among children and pregnant women across 15 countries using standardized national surveys. Am J Trop Med Hyg. 2009;80(2): 209–214 19190215

[24] Roll Back Malaria Partnership. The global malaria action plan for a malaria-free world: executive summary. Geneva: World Health Organizaton/Roll Back Malaria Partnership; 2008 Available from: http://www.rollbackmalaria.org/gmap/0-5.html

[25] Webster J Kayentao K Bruce J Diawara SI Abathina A Haiballa AA Prevention of malaria in pregnancy with intermittent preventive treatment and insecticide treated nets in Mali: a quantitative health systems effectiveness analysis. PLoS One. 2013;8(6): e67520 10.1371/journal.pone.0067520 23840729 PMC3695962

[26] Kilian A Koenker H Paintain L Estimating population access to insecticide-treated nets from administrative data: correction factor is needed. Malar J. 2013;12:259 10.1186/1475-2875-12-259 23890257 PMC3726288

